# Electron Transfer to Hydroxylase through Component Interactions in Soluble Methane Monooxygenase

**DOI:** 10.4014/jmb.2201.01029

**Published:** 2022-02-05

**Authors:** Chaemin Lee, Yunha Hwang, Hyun Goo Kang, Seung Jae Lee

**Affiliations:** 1 Department of Chemistry, Jeonbuk National University, Jeonju 54896, Republic of Korea; 2 Department of Neurology, Research Institute of Clinical Medicine of Jeonbuk National University and Biomedical Research Institute of Jeonbuk National University Hospital, Jeonju 54907, Republic of Korea; 3 Institute for Molecular Biology and Genetics, Jeonbuk National University, Jeonju 54896, Republic of Korea

**Keywords:** Soluble methane monooxygenase (sMMO), electron transfer, reductase, bacterial multicomponent monooxygenase (BMM), hydroxylation

## Abstract

The hydroxylation of methane (CH_4_) is crucial to the field of environmental microbiology, owing to the heat capacity of methane, which is much higher than that of carbon dioxide (CO_2_). Soluble methane monooxygenase (sMMO), a member of the bacterial multicomponent monooxygenase (BMM) superfamily, is essential for the hydroxylation of specific substrates, including hydroxylase (MMOH), regulatory component (MMOB), and reductase (MMOR). The diiron active site positioned in the MMOH &alpha;-subunit is reduced through the interaction of MMOR in the catalytic cycle. The electron transfer pathway, however, is not yet fully understood due to the absence of complex structures with reductases. A type II methanotroph, *Methylosinus sporium* 5, successfully expressed sMMO and hydroxylase, which were purified for the study of the mechanisms. Studies on the MMOH-MMOB interaction have demonstrated that Tyr76 and Trp78 induce hydrophobic interactions through &pi;-&pi; stacking. Structural analysis and sequencing of the ferredoxin domain in MMOR (MMOR-Fd) suggested that Tyr93 and Tyr95 could be key residues for electron transfer. Mutational studies of these residues have shown that the concentrations of flavin adenine dinucleotide (FAD) and iron ions are changed. The measurements of dissociation constants (K_d_s) between hydroxylase and mutated reductases confirmed that the binding affinities were not significantly changed, although the specific enzyme activities were significantly reduced by MMOR-Y93A. This result shows that Tyr93 could be a crucial residue for the electron transfer route at the interface between hydroxylase and reductase.

## Introduction

The greenhouse gases, including carbon dioxide (CO_2_) and methane (CH_4_), are important contributors to global warming, which has detrimental consequences on ecological systems [1-3]. Methane gas is considered the most harmful molecule because of its high heat capacity, compared to that of carbon dioxide [4-9]. The hydroxylation of methane requires high temperature and pressure as C–H activation requires high energy (105 kcal/mol) [10]. This chemical reaction can be performed through soluble methane monooxygenases (sMMOs) under mild ambient pressure and temperature conditions. Understanding the mechanisms of the enzymatic activities will enhance its applications in the field of biotechnology, although it has a complicated system as a member of the bacterial multicomponent monooxygenase (BMM) superfamily [2, 3, 11-15]. Soluble MMO has hydroxylase (MMOH), a regulatory component (MMOB), and reductase (MMOR) for the conversion of methane to methanol; therefore, understanding the activation mechanisms is of utmost importance.

**Fig. 1:**
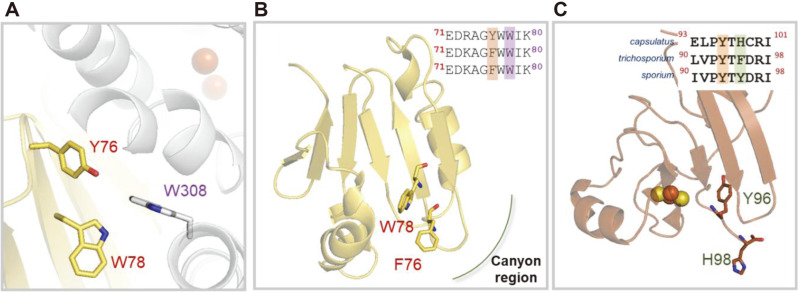
Multicomponent interactions between hydroxylase and MMOB or MMOR for the catalytic reactions. (**A**) Hydrophobic interactions are generated between MMOH (gray) and MMOB (yellow) from *Methylococcus capsulatus* (Bath) (PDB: 4GAM). The orange balls present diiron in active sites. (**B**) Core region of MMOB from *Methylosinus trichosporium* OB3b has Phe76 and Trp78 that generate hydrophobic interactions (PDB: 2MOB). (**C**) Ferredoxin domain of MMOR (MMOR-Fd) is a putative binding site to the surface of MMOH α-subunit for electron transfers from *M. capsulatus* (PDB: 1JQ4). *Capsulatus*, *trichosporium* and *sporium* represent *M. capsulatus* (Bath), *M. trichosporium* OB3b, and *Methylosinus sporium* 5, respectively.

The dimeric form of MMOH has an αβγ protomer of approximately 250 kDa, and long or short α-helices are the major secondary structures of these macromolecules [16-19]. Through structural studies, it has been confirmed that the diiron active sites (Fe-Fe) are buried around 12 Å from the surface of the MMOH α-subunit, and four Glu and two His residues coordinate with diiron for the catalytic cycle [18]. These six residues are positioned in the four-helix bundle in the α-subunit of MMOH, especially the B, C, E, and F helices. The coordination of the diiron active sites is changed by the conformational changes of these four-helix bundles [20]. The dimerization of α– and β– subunits is critical for catalytic activities and forms a canyon region that is important for the interactions of MMOB and MMOD [20, 21]. The complex structures of MMOH-MMOB and MMOH-MMOD revealed that MMOB and MMOD bind this canyon region to induce allosteric effects in diiron active sites. This event finally induces structural modifications at the inner sphere of MMOH for catalytic activities, and these allosteric effects further regulate substrate ingress and product release. The reported MMOH-MMOB complex from *Methylococcus capsulatus* Bath showed that the regulatory component generates hydrogen bonds and hydrophobic packing on the surface of MMOH [20]. The indole moiety from Trp308 on MMOH generates hydrophobic interactions with MMOB, including Tyr76 and Trp78. This π-stacking is considered an efficient interaction between the core regions of MMOB (Fig. 1A). MMOBs can be classified into three regions based on their structures [12, 17, 20, 22-29]. The N-terminal region (1-35) does not have a specific secondary structure in NMR studies, but it has a short α-helix through specific hydrogen bonds to the surface of MMOH [24, 28]. Enzyme activity studies and biophysical investigations have shown that the N-terminus is essential for the catalytic activity of sMMO [20, 29]. The specific enzyme activity diminishes more than 200 times in the absence of a regulatory component. The core region (36–109) has a well-folded β-strand and its globular shape faces the canyon region of the hydroxylase. The structure and specific functions of the C-terminus (110–128) have not yet been reported.

Electron transfer from MMOR to diiron active sites in MMOH is a necessary step in the catalytic cycle of sMMO [22, 30-34]. The reduction of the active site from diferric (Fe^III^-Fe^III^) to diferrous (Fe^II^-Fe^II^) is performed through electron transfer, which produces diverse intermediates through O_2_ activation [35-38]. The reported mechanisms of sMMO suggest that O_2_ activation is crucial for C–H activation as methane is only oxidized in the high-valent Q-intermediate (Fe^IV^-Fe^IV^) [14, 39-41]. The MMOH-MMOB complex provides detailed mechanisms of substrate pathways and coordinated environments of active sites in MMOH [20]. The conformational shifts and mechanisms of these active sites are well explained by the interaction between MMOH and MMOB. The hydrophobic interactions, including Tyr76 and Trp78 of MMOB, induce π-π stacking and further changes in MMOH (Fig. 1A). These hydrophobic interactions are efficient for MMOH because other components can share these binding sites with low-energy barriers compared with those of hydrogen bonds. The structure of MMOR has not yet been reported, although information for each domain, ferredoxin domain (MMOR-Fd) and FAD/NADH-binding domain (MMOR-FAD), has been reported [22, 30-34]. These studies proposed that NADH transfers hydride (H-) to MMOR-FAD and an electron will further push it to MMOR-Fd for the reduction of diiron active sites. The superimposed structure between MMOR-Fd and the core region of MMOB has a relatively small root-mean-square deviation (r.m.s.d. = 5.508). The core region of MMOB from the type II methanotroph, *Methylosinus trichosporium* OB3b, has two aromatic residues, Phe76 and Trp78, for hydrophobic interactions (Fig. 1B). The superimposed structure of MMOR-Fd has an aromatic residue, Tyr96, in this hydrophobic interaction area [30, 34]. In addition, His98 is positioned in the solvent-accessible area and these residues are considered to be putative binding residues to MMOH from *M. capsulatus* Bath (Fig. 1C).

The electron transfer pathway has not yet been elucidated, although it is one of the most important factors for catalytic activity. As described in Figs. 1B and 1C, putative binding residues from *Methylosinus sporium* 5 were investigated in this study. Two domains of MMOR have different cofactors including FAD in MMOR-FAD and irons in MMOR-Fd. Previous studies have reported that key residues in MMOR cause changes in the concentration of cofactors [6]. The molar ratio of iron to MMOR changes due to the loss of the secondary or tertiary structure of the ferredoxin domain. FAD, the other cofactor, is positioned between the NADH-binding domain and the FAD-binding domain. Mutational studies show the FAD-binding domain causes a change in the binding of this cofactor [6]. In this study, Tyr93 and Tyr95 from *M. sporium* 5 were mutated to Ala to investigate possible structural and functional changes [34]. The concentration of FAD is changed by the mutations of these residues, which indicates structural modification by these mutations. This study also demonstrates that structural modifications of MMOR-Y93A affect catalytic activity, although they do not influence the binding affinity to MMOH. These results will provide crucial information for the understanding of electron transfer in the sMMO system for final applications in biotechnology.

## Materials and Methods

### General Materials

Chemicals were purchased from Sigma-Aldrich (USA). Synthetic nucleotides for the expression and purification of MMOB, MMOR, and mutated MMORs were purchased and sequenced by Cosmogenetech (Korea). Hydroxylase (MMOH) of soluble methane monooxygenase (sMMO) was expressed and purified from *M. sporium* strain 5 (ATCC35069), which was purchased from the American Type Culture Collection (ATCC). BL21(DE3) and pET30a (+) were purchased from EMD Millipore. DH5α (DE3) cells were purchased from New England Biolabs (USA). Propylene (99.5%) and methane gas (99.9%) for enzyme activity measurements and cell culture were purchased from Hankook Gas (Korea). Protein purification was performed using an ATKA Pure 25 L Fast Protein Liquid Chromatography (FPLC) System (GE Healthcare Life Science, USA). Optical absorption was measured using a UV-visible spectrometer (Agilent Technologies, Cary 60, USA). Cells and proteins were harvested by centrifugation using centrifuges from Beckman Coulter (Allegra X-15R, USA) and Hanil Science (Combi 514R, Korea). The expression and purification of MMOH and MMOB were performed according to protocols described in previous reports [6].

### Expression and Purification of MMORs

For the mutation of Tyr93 or Tyr95 to Ala in MMOR, the primers for site-directed mutagenesis were designed as follows: MMOR-Y93A forward primer: 5’-TCGTGCCCGCCACCTATGATC and MMOR-Y95A forward primer: 5’-TGCCCTACACCGCTGATCGCATC. Mutations were performed using a Q5 site-directed mutagenesis kit (NEB, England). The plasmids were transformed into DH5α competent cells and nucleotides were extracted for sequencing. The sequenced plasmids were transformed into BL21(DE3) competent cells and used for the expression of MMORs.

MMORs in BL21(DE3) were incubated in LB broth (Lennox, 50 μg/ml kanamycin) at 200 rpm at 37°C. When the optical density at 600 nm (OD_600_) reached 0.8, ammonium iron (II) sulfate (0.5 mM) was added to the culture to induce MMORs with 0.5 mM isopropyl β-D-1-thiogalactopyranoside (IPTG) at 25°C and 200 rpm. Cell pellets were harvested by centrifugation at 11,355 ×*g* for 20 min at 4°C. Cells were lysed through a sonicator (Sonics, CV334) with 25% output (15 s on and 45 s off) in lysis buffer (25 mM 3-(N-morpholino)propanesulfonic acid (MOPS), 50 mM NaCl, 2.0 mM ammonium iron(II) sulfate, 5.0 mM MgCl_2_, 2.0 mM L-Cysteine, 8.0 mM mercaptoacetic acid sodium salt, 0.01 μl/ml DNase I, and 0.002 mg/ml phenylmethylsulfonyl fluoride (PMSF) at pH 6.5. The lysed mixture was centrifuged at 15,922 ×*g* for 50 min (Hanil Science, Supra 22 K) to obtain the supernatant. Eluates were filtered using a 0.22 μM membrane filter (Satorius 16534).

The purifications were performed using three-step processes to obtain more than 95% purity. The lysed supernatant was applied to a Q-sepharose column (XK 26/400, GE Healthcare) incubated with equilibrium buffer (25 mM MOPS, 1 M NaCl, 200 μM ammonium iron (II) sulfate, 2.0 mM L-Cysteine, 8.0 mM mercaptoacetic acid sodium salt, 10% glycerol at pH 6.5) with 0–1,000 mM NaCl gradient. The eluates were confirmed using 10%sodium dodecyl sulfate-polyacrylamide gel electrophoresis (SDS-PAGE) with Coomassie Brilliant Blue staining. Target-containing eluates were concentrated through a 30 kDa cut-off membrane filter (Merck Millipore, UFC903024, USA) at 3,200 rpm at 4°C. The concentrated eluates were applied to a Superdex 75 (XK 26/700, GE Healthcare) equilibrated with Buffer A (25 mM MOPS, 50 mM NaCl, 1 mM dithiothreitol (DTT), and 10%glycerol at pH 6.5) under isocratic conditions. The eluates were concentrated with a 30 kDa cut-off membrane filter and applied to a final Q-Sepharose column equilibrated with Buffer A to obtain MMORs with ≥ 95% purity.

### Measurements of Cofactor Contents in MMORs

The UV-Vis absorption spectra were monitored using a Cary 60 (Agilent Technologies) in a cuvette (Helma Analytics, Germany) to calculate the concentration of Fe and FAD in MMOR. The concentration of iron in the MMOR was calculated using the ferrozine assay described elsewhere [6]. The FAD concentration was measured at 394 and 458 nm through the calibration of the [2Fe-2S]-cluster peaks, including 332, 418, and 467 nm [6, 22, 31, 32].

### Measurements of Specific Enzyme Activity

The enzyme activities were measured using a UV-Vis spectrometer by monitoring β-nicotinamide adenine dinucleotide, reduced disodium salt hydrate (NADH, Sigma-Aldrich) consumption at 340 nm. MMOH (1 μM in 500 μl) and 2 equiv. of MMOB were incubated with reaction buffer (25 mM phosphate and 50 mM NaCl) at pH 7.0 in the presence of propylene and oxygen gases. The reactions were performed in the presence of 0.5 or 1.0 equiv. of MMORs and monitored at 340 nm every second with the addition of NADH (50 nmol) for 1 min.

### Measurements of Binding Affinity Between MMOH and MMORs

Tryptophan quenching was measured using a spectrofluorometer (JASCO, FP-8300, Japan) with a cuvette (JASCO, J/3 type material Q) with 25 mM MOPS, 50 mM NaCl, and 1 mM DTT at pH 6.5. The final concentration of MMOH was 0.32 μM (800 ml). The excitation (E_x_ = 282 nm) and emission (E_m_ = 336 nm) spectra were also determined [6]. MMORs were added to cuvettes containing MMOH from 0 to 110 equivalents to achieve titration curves, and the dissociation constants (K_d_s) were obtained using the 1:2 binding model (R^2^ > 0.99) in OriginPro [6].

## Results and Discussion

### Characterization of MMOR-Y93A and MMOR-Y95A

**Fig. 2:**
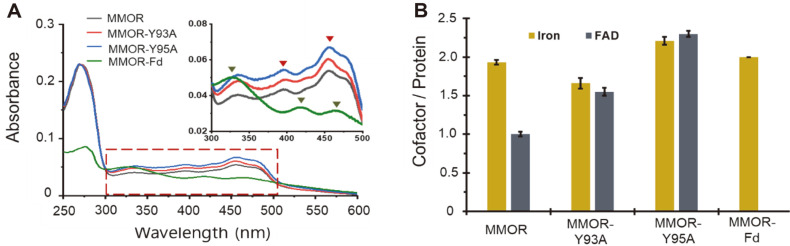
Mutated MMOR influences the contents of cofactor from *M. sporium* 5. (**A**) UV-visible spectrum of purified MMOR (dark green), MMOR-Y93A (red), MMOR-Y95A (cyan), and MMOR-Fd (green). The specific peaks from ferredoxin domain are indicated as green reverse-triangles (332, 418, and 467 nm) and FAD domain is indicated as red reversetriangles (394 and 458 nm). (**B**) Molar ratio of cofactor including iron (dark yellow) and FAD (dark green). All experiments were performed at least three times (ave ± std).

The target mutations, including MMOR-Y93A and MMOR-Y95A, were successfully expressed in *E. coli* and the expressed enzymes were purified through a three-step purification method to yield more than 95% purity. Reported results showed that mutations in the MMOR FAD-binding domain (MMOR-FAD) cause changes in not only the molar ratio of FAD/MMOR but also the molar ratio Fe/MMOR [6]. Monitoring these events through UV-Vis spectrometry reveals that subtle changes in MMOR can cause dramatic modifications in the structure (Fig. 2A). The MMOR has specific absorptions at 332, 418, and 467 nm owing to the [2Fe-2S]^+^ cluster in MMOR-Fd (green triangles in Fig. 2A inlet). As a reference, MMOR-Fd, the N-terminus position of MMOR (1-98), was successfully expressed and purified to monitor its absorption. The absorption of MMOR-Fd did not show any specific absorption from FAD, but mutated MMORs show different patterns compared with that of wild-type MMOR. These results demonstrate that single-point mutations in MMORs influence their structure and cofactor interactions.

Ferrozine assay shows that MMOR-Y93A has 1.7 ± 0.1 Fe/protein (Fig. 2, Table S1), although the content of MMOR is 1.9 ± 0.1 Fe/protein. This result indicates that the coordination of MMOR-Fd is slightly influenced by mutation. As an important cofactor for electron transfer in reductase, the number of FADs is increased in these mutations. These modifications of cofactors can be explained by the structural changes in reductase, which can be explained as non-specific interactions. Mutational studies of MMOR-FAD from the first X-ray structure of MMOR-FAD from *M. sporium* 5 indicate that mutations in MMOR-FAD result in different iron and FAD contents due to structural alterations [6, 30]. This study confirms that Tyr160 is a key residue for electron flow in MMOR-FAD, since its catalytic activity is completely abolished by mutations [6]. Aromatic compounds such as Tyr can be used as stepping stones for electron jumping, but specific enzyme activities were not recovered from MMOR-Y160F. In this study, the mutation of MMOR-Y93A has two FADs like MMOR-Y160F as monitored in different domains. These patterns were also monitored on the other mutation, MMOR-Y95A, since the ferrozine assay recognized two irons and two FADs per purified reductase. Our findings show that a single mutation of MMOR in some residues can cause structural changes that mediate non-specific interactions to cofactors.

### Activities of Mutated MMORs in Ferredoxin Domain

**Fig. 3:**
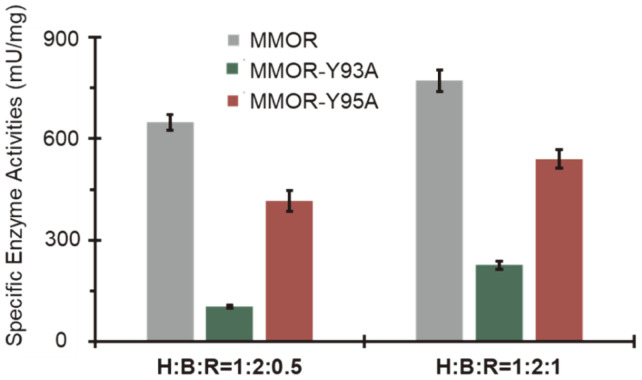
Specific enzyme activities of MMOR and mutated MMOR. Enzyme activities of MMOR-Y93A were reduced. H : B : R indicates the molar ratio of MMOH, MMOB, and MMOR (gray) or mutated MMOR including MMOR-Y93A (green) and MMOR-Y95A (red). All experiments were performed at least three times (ave ± std).

Previous reports on mutational studies of MMOR-FAD have suggested that NADH consumption is critically influenced by the MMOR [6, 22]. Specific enzyme activities were measured to evaluate electron transfer in the presence of 0.5 equiv. or 1.0 equiv. of MMORs. The highly purified MMOH from *M. sporium* 5 shows significant specific enzyme activities (648.4 mU/mg in 0.5 equiv. MMOR and 771.0 mU/mg in 1.0 equiv. MMOR) compared with those from type X methanotrophs (Fig. 3, Table S2). The initiation of electron transfer is generated by the binding of NADH [22, 29-34]. Afterward, FAD accepts electrons inside the reductase. The abnormal binding of FAD to MMOR retards electron transfer from NADH to the final destination, the diiron active site, through MMOR.

The activity of MMOR-Y93A was dramatically reduced, although it had 1.7 Fe/protein and 2.0 FAD/protein (Fig. 3, Table S2). The activity of MMOR-Y93A diminished by more than 80% in the presence of 0.5 equiv. mutated MMOR, while the specific enzyme activity of MMOR-Y95A reduced by approximately 30% under identical conditions. For catalytic activities, Y93A is considered an important residue for electron transfer. The specific enzyme activities slightly increased in the presence of 1.0 equiv. MMORs, and MMOR-Y93A showed approximately 70% decrease in activity. MMOR-Y95A showed a 23% decrease in MMOR activity.

The diminished specific enzyme activity from MMOR-Y93A can be explained by the structural modification or blocking of the electron transfer routes in MMOR. Previous results suggested that electron transfer in MMOR could be abolished by the side-chain from Tyr160 in the MMOR-FAD domain [6]. This can be explained by the elimination of the hydrogen bonding of hydroxyl groups in the Tyr residue. The other mutation, MMOR-Y160F, in the FAD-binding domain, could not recover its electron transfer activities. This result directly shows that hydrogen bonds arrange the position of the phenyl group in Tyr160, and this side-chain is necessary for its electron transfer route. The electron transfer of MMOR is initiated from NADH to MMOR-FAD and finally reaches MMOR-Fd to deliver it to the diiron active site in MMOH. Tyr93 is considered a crucial residue for electron transfer. Another possibility is the disruption of the binding between MMOH and MMOR-Y93A.

### Binding Affinities Between Hydroxylase and MMORs

The mutational study of the FAD-binding domain, MMOR-Y160F, indicated that binding affinity is also affected by the possible modification of its structure. An interesting aspect of this event is the modification of the FAD-binding domain, which influences the dissociation constant (K_d_) between MMOH and MMOR-Y160F. It was postulated that modification of MMOR-Y160F does not affect the binding to MMOH, because MMOR-Fd directly interacts with the α-subunit [6, 22, 31, 32]. The mutations of Tyr160 to Ala, however, do not induce any interactions with MMOH, and the loss of binding affinity is the major reason for electron blocking to diiron active sites. The structural modification of Tyr160 can hamper the binding to the α-subunit of MMOH, and this event breaks complex binding. To understand the structural effects in electron transfer of Tyr160, the specific enzyme activity and cofactor contents were investigated using Y160F. This previous study confirmed that the hydrogen bond of Tyr160 is crucial because Y160F blocks electron transfer from NADH to the diiron active site.

**Fig. 4:**
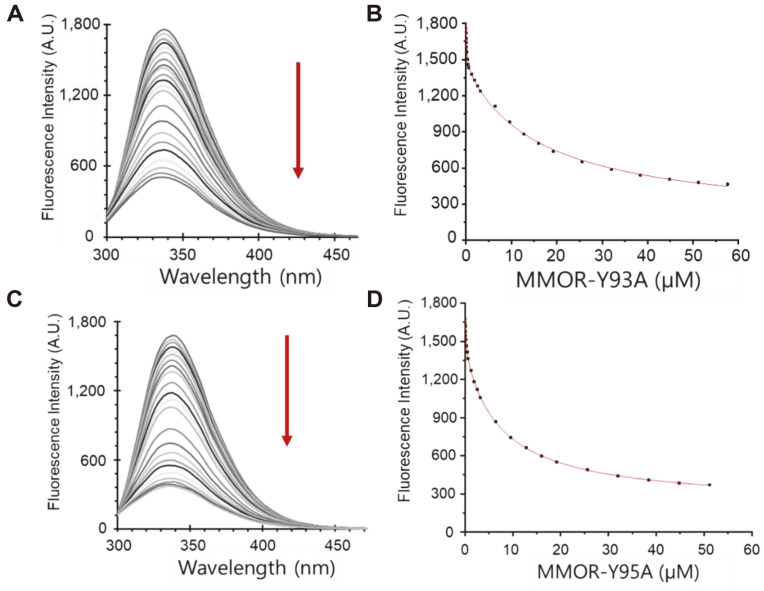
Measurements of binding affinities between MMOH and mutated MMOR including Y93A and Y95A. The fluorescence intensities were reduced by the addition of mutated MMORs. (**A**) Fluorescence intensities reduced following the addition of MMOR-Y93A. (**B**) Decreased fluorescence intensities between MMOH and MMOR-Y93A by the addition of MMOR-Y93A were fitted through 1:2 binding. (**C**) Fluorescence intensities reduced following the addition of MMOR-Y95A. (**D**) Decreased fluorescence intensities between MMOH and MMOR-Y95A were fitted through 1:2 binding model.

In this study, the specific enzyme activity of MMOR-Y93A indicated that electron transfer was blocked. The binding affinity needs to be investigated to understand whether Tyr93 blocks the electron directly by positioning on its transfer route or hampering of electron delivery due to the dissociation between MMOH and MMOR-Y93A. The dissociation constants (K_d_s) between MMOH and MMORs were investigated by measuring tryptophan quenching, and the results show that K_d1_ and K_d2_ were not significantly influenced compared with those of MMOR (Fig. 4, Table S3). It is thought that reduced MMOR will bind oxidized hydroxylase to transfer electrons, although the details of these mechanisms are not yet fully understood because of the absence of a complex structure [6, 12, 14, 19, 22, 30, 32, 40, 42-44]. The K_d1_ of the reduced MMOR to oxidized MMOH was approximately two times higher, but K_d2_ was almost similar. The first dissociation constant (K_d1_) between MMOH and MMOR-Y93A was 0.15 ± 0.01 μM, and this demonstrated that MMOR-Y93A did not affect the interaction with hydroxylase. This result also implies that the structure of the binding region in MMOR was not changed by the Y93A mutation in MMOR, although its specific enzyme activity was reduced sharply. The binding and activity assays confirmed that MMOR-Y93A blocked the electron transfer route without structural modification. The second dissociation constant (K_d2_) of MMOR-Y93A increased slightly (16.96 ± 0.37 μM) because the first binding of MMOR-Y93A induced structural modification in MMOH allosterically. The electron transfer of MMOR-Y95A was retarded slightly compared with that of MMOR, but it showed similar dissociation constants with 0.21 ± 0.03 μM (K_d1_) and 7.97 ± 0.33 μM (K_d2_), respectively.

The catalytic cycle of a BMM superfamily is a crucial issue, because electron transfer to the diiron active site is essential for achieving a diferrous state for O_2_ activation [14, 40, 45, 46]. The electron transfer in these enzymatic systems is quite complicated because of the interactions of the components, including reductase and hydroxylase. In addition, the hydroxylase of sMMO is more than 250 kDa and consists of six polypeptides, and reductase, with a large molecular weight (39 kDa). Previous research confirmed that electron transfer from MMOR to MMOH is an elaborate route, and the elucidation of these pathways requires diverse methods of enzymology [6, 22, 31, 32]. This study confirms that one important residue, Tyr93, can be positioned at the interface between MMOR and MMOH. Previous reports on Tyr160 have shown that enzymatic activities were completely abolished and that MMOR-Y160A or MMOR-Y160F could not bind to hydroxylase with a significant association constant [6]. The putative binding residues, including Tyr93 and Tyr95, were mutated to understand their possible roles in the electron transfer pathway. Both mutations generate non-specific interactions because mutated MMOR has more than one equivalent FAD. The specific enzyme activity of MMOR-Y93A was blocked, and its binding affinities were not influenced. These results demonstrate that Tyr93 is positioned on the electron transfer route. In this study, we identified one residue positioned in the electron pathway in MMOR, especially in the ferredoxin domain. Tyr160 is positioned in the FAD-/NADH-binding domain, and the mutations of MMOR-Y160A induce conformational changes in other MMOR residues [6]. MMOR-Y93A generated a similar pattern of structural modifications. The mutation is induced in the ferredoxin domain, but it generates changes in the cofactor in MMOR-FAD. This study provides information about the electron route and it can be further elucidated with more studies on the MMOR and MMOH complex, which is essential for catalytic activities.

## Supplemental Materials

Supplementary data for this paper are available on-line only at http://jmb.or.kr.
