# Supplementary material for: Electron Transfer to Hydroxylase through Component Interactions in Soluble Methane Monooxygenase
Source: J Microbiol Biotechnol. 2022 Feb 5;32(3):287–93. doi: 10.4014/jmb.2201.01029 (PMC9628860; doi:10.4014/jmb.2201.01029)
Supplement: Supplementary file 1 [file jmb-32-3-287-supple.pdf]

J. Microbiol. Biotechnol.
https://doi.org/10.4014/jmb.2201.01029

SUPPLEMENTAL TABLES

TABLE S1. Molar ratio of iron or FAD to MMOR and mutated MMOR. All experiments were performed at least three times (ave ± std).

	[Fe]	[FAD]
MMOR	1.9 ± 0.0	1.0 ± 0.0
MMOR-Y93A	1.7 ± 0.1	1.6 ± 0.1
MMOR-Y95A	2.2 ± 0.1	2.3 ± 0.0
MMOR-Fd	2.0 ± 0.0	N.D.

*N.D. represents not detected.

TABLE S2. Measurements of specific enzyme activities (mU/mg) of MMOH with 2 equiv. of MMOB and MMOR or mutated MMOR including MMOR-Y93A, and MMOR-Y95A. The activities were measure by the molar ratio of 0.5 equiv. or 1.0 equiv. of reductases. All experiments were performed at least three times (ave ± std).

	Specific enzyme activity (mU/mg)	
	H:B:R=1:2:0.5	H:B:R=1:2:1
MMOR	648.4 ± 22.8	771.0 ± 32.4
MMOR-Y93A	103.1 ± 4.2	226.3 ± 11.9
MMOR-Y95A	415.6 ± 30.3	539.9 ± 27.1

TABLE S3. Measurements of binding affinities between MMOH and MMORs or mutated MMOR. The 1:2 binding model is applied for the fitting between MMOH and reductases. All experiments were performed at least three times (ave ± std).

	K_{d1} (μM)	K_{d2} (μM)
Oxidized MMOR	0.38 ± 0.04	6.72 ± 0.69
Reduced MMOR	0.17 ± 0.04	6.87 ± 0.69
MMOR-Y93A	0.15 ± 0.01	16.96 ± 0.37
MMOR-Y95A	0.21 ± 0.03	7.97 ± 0.33
